# Evaluating Simplified IVIM Diffusion Imaging for Breast Cancer Diagnosis and Pathological Correlation

**DOI:** 10.3390/diagnostics15162033

**Published:** 2025-08-14

**Authors:** Abdullah Hussain Abujamea, Salma Abdulrahman Salem, Hend Samir Ibrahim, Manal Ahmed ElRefaei, Areej Saud Aloufi, Abdulmajeed Alotabibi, Salman Mohammed Albeshan, Fatma Eliraqi

**Affiliations:** 1 Department of Radiology and Medical Imaging, College of Medicine, King Saud University (KSU), Riyadh 11461, Saudi Arabia; abujamea@ksu.edu.sa (A.H.A.);; 2 Royal Commission Medical Centre, Yanbu 46457, Saudi Arabia; 3 Alahrar Teaching Hospital, General Organization for Teaching Hospitals and Institutes, Cairo 11697, Egypt; 4 Department of Radiological Sciences, College of Applied Medical Sciences, King Saud University (KSU), P.O. Box 145111, Riyadh 11433, Saudi Arabia; 5 Department of Radiological Sciences, College of Applied Medical Sciences, King Saud bin Abdulaziz University for Health Sciences, Riyadh 11426, Saudi Arabia; otaibiabdulm@ksau-hs.edu.sa; 6 King Abdullah International Medical Research Centre, National Guard Health Affairs, Riyadh 11426, Saudi Arabia; 7 The General Organization for Teaching Hospitals and Institutes, (National Institute of Neurology and Urology), Cairo 11765, Egypt

**Keywords:** breast cancer, magnetic resonance imaging, intravoxel incoherent motion, diffusion-weighted imaging

## Abstract

**Background/Objectives:** This study aimed to evaluate the diagnostic performance of simplified intravoxel incoherent motion (IVIM) diffusion-weighted imaging (DWI) parameters in distinguishing malignant from benign breast lesions, and to explore their association with clinicopathological features. **Methods:** This retrospective study included 108 women who underwent breast MRI with multi-b-value DWI (0, 20, 200, 500, 800 s/mm^2^). Of those 108 women, 73 had pathologically confirmed malignant lesions. IVIM maps (ADC_map, D, D*, and perfusion fraction f) were generated using IB-Diffusion™ software version 21.12. Lesions were manually segmented by radiologists, and clinicopathological data including receptor status, Ki-67 index, cancer type, histologic grade, and molecular subtype were extracted from medical records. Nonparametric tests and ROC analysis were used to assess group differences and diagnostic performance. Additionally, a binary logistic regression model combining D, D*, and f was developed to evaluate their joint diagnostic utility, with ROC analysis applied to the model’s predicted probabilities. **Results:** Malignant lesions demonstrated significantly lower diffusion parameters compared to benign lesions, including ADC_map (*p* = 0.004), D (*p* = 0.009), and D* (*p* = 0.016), indicating restricted diffusion in cancerous tissue. In contrast, the perfusion fraction (f) did not show a significant difference (*p* = 0.202). ROC analysis revealed moderate diagnostic accuracy for ADC_map (AUC = 0.671), D (AUC = 0.657), and D* (AUC = 0.644), while f showed poor discrimination (AUC = 0.576, *p* = 0.186). A combined logistic regression model using D, D*, and f significantly improved diagnostic performance, achieving an AUC of 0.725 (*p* < 0.001), with 67.1% sensitivity and 74.3% specificity. ADC_map achieved the highest sensitivity (100%) but had low specificity (11.4%). Among clinicopathological features, only histologic grade was significantly associated with IVIM metrics, with higher-grade tumors showing lower ADC_map and D* values (*p* = 0.042 and *p* = 0.046, respectively). No significant associations were found between IVIM parameters and ER, PR, HER2 status, Ki-67 index, cancer type, or molecular subtype. **Conclusions:** Simplified IVIM DWI offers moderate accuracy in distinguishing malignant from benign breast lesions, with diffusion-related parameters (ADC_map, D, D*) showing the strongest diagnostic value. Incorporating D, D*, and f into a combined model enhanced diagnostic performance compared to individual IVIM metrics, supporting the potential of multivariate IVIM analysis in breast lesion characterization. Tumor grade was the only clinicopathological feature consistently associated with diffusion metrics, suggesting that IVIM may reflect underlying tumor differentiation but has limited utility for molecular subtype classification.

## 1. Introduction

Breast cancer continues to be the most frequently diagnosed cancer and a major cause of cancer-related mortality among women globally [1]. Achieving an accurate diagnosis is critical for selecting appropriate therapeutic strategies and improving clinical outcomes [2]. Dynamic contrast-enhanced MRI (DCE-MRI), has been widely recognized for its high sensitivity in detecting and characterizing breast abnormalities [3,4,5]. However, the specificity of DCE-MRI remains relatively low, which can result in unnecessary biopsies [5]. Diffusion-weighted imaging (DWI), has gained traction as a complementary tool by offering quantitative measures such as the apparent diffusion coefficient (ADC) [6,7,8,9]. The development of intravoxel incoherent motion (IVIM) analysis has further advanced the capability of DWI by distinguishing between molecular diffusion and microvascular perfusion. IVIM enables estimation of key parameters, including the true diffusion coefficient (D), perfusion fraction (f), and pseudodiffusion coefficient (D*) [10]. Studies have shown that evaluating D instead of ADC yields higher diagnostic accuracy when used both independently and alongside DCE-MRI [9,11]. This is attributed to the fact that malignant breast lesions typically exhibit restricted diffusion and elevated perfusion, causing ADC values to overlap with those of benign lesions, while D values remain distinct [12,13,14,15,16,17]. Notably, the combined assessment of D and f has demonstrated improved diagnostic performance as a stand-alone technique, although this benefit is less apparent when used in conjunction with DCE-MRI [5,18].

It is worth noting that traditional IVIM analysis involves fitting all three parameters (D, D*, and f) using non-linear regression models. However, this method typically requires a large number of b-values and extended acquisition times [19], which can compromise fitting stability, reproducibility, and parameter reliability, especially in tissues with inherently low perfusion such as normal fibroglandular or benign tissue [18,19,20,21,22]. To address these limitations, a simplified IVIM approach has been proposed, which assumes a negligible contribution of the pseudodiffusion component at higher b-values. This technique allows for direct, stable calculation of D′, f′, and D*′ using fewer b-values, resulting in shorter scan durations and improved parameter robustness [5,22,23,24,25].

Recent studies have highlighted the diagnostic strength of simplified IVIM DWI in distinguishing benign from malignant breast lesions. Diagnostic accuracy using combined D1′ and f1′ values within perfusion hotspots has been reported at approximately 93.7%, notably higher than the 86.9% accuracy associated with ADC alone [5,23]. When used independently, simplified IVIM DWI offers a non-contrast alternative with diagnostic performance comparable to, and in some cases approaching, that of DCE-MRI, depending on lesion type and image quality [23].

Accordingly, the present study aims to assess the diagnostic performance of simplified IVIM parameters (use of a reduced number of b-values during acquisition and analysis, which allows for a more practical and time-efficient assessment while maintaining diagnostic utility) in differentiating malignant from benign breast lesions. In addition, the study evaluates the utility of IVIM metrics in characterizing molecular breast cancer subtypes and explores their associations with key clinicopathological features.

## 2. Materials and Methods

### 2.1. Participants

This retrospective study received approval from the local institutional review board at King Khalid University Hospital (KKUH) (IRB# 23/0209). Between August 2021 and September 2023, a total of 108 women with a mean age of 46.2 years (SD = 13.2) underwent standard breast imaging protocols incorporating multi-b-value DWI. Of those 108 women, 73 had pathologically confirmed malignant lesions.

### 2.2. MRI Protocol

MRI examinations were conducted using a 1.5 T MRI scanner (Optima MR450w, GE-Healthcare, Milwaukee, WI, USA) with a dedicated breast coil. Patients were positioned prone to facilitate natural breast placement within the coil. The imaging protocol is described in Table 1.

### 2.3. Image Analysis and Post-Processing

ADC, D, D*, and f values were calculated using the OsiriX-plugin IB-Diffusion™ (version 21.12) Imaging Biometrics, Elm Grove, WI, USA) [26]. IVIM parameters were calculated based on the segmented bi-exponential intravoxel incoherent motion (IVIM) model, as originally described by Le Bihan et al., 1989 [27]. The model is expressed by the following equation:

Sb/S_0_ = (1 − f) · exp(–bD) + f · exp[–b (D* + D)]

where Sb is the signal intensity at a given diffusion weighting (b-value), and S_0_ is the signal intensity without diffusion weighting (b = 0). The D value represents the true diffusion coefficient, reflecting the pure molecular diffusion of water in the extracellular and extravascular space, independent of perfusion effects. The D* value corresponds to the pseudo-diffusion coefficient, which is associated with perfusion-related effects caused by microcirculation within the capillary network. The f value represents the perfusion fraction, indicating the relative contribution of perfusion to the overall diffusion signal [12].

**Figure 1:**
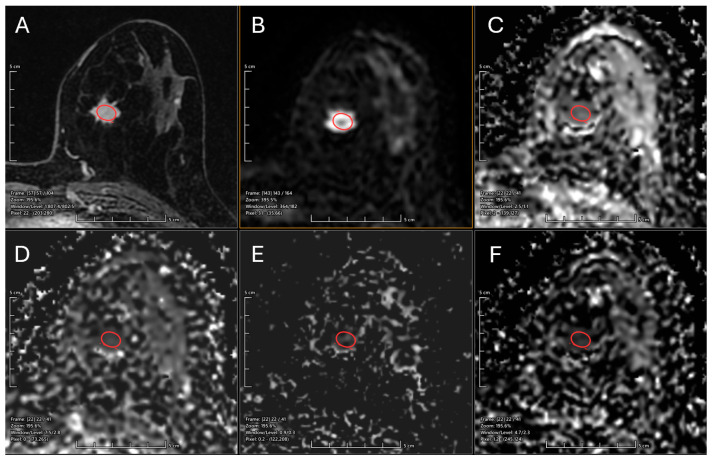
Examples of Region of Interest (Red circle) placement in axial images from a malignant breast lesion. (**A**) Contrast-enhanced T1-weighted image; (**B**) diffusion-weighted (DW) image with a b-value of 800 s/mm^2^; (**C**) apparent diffusion coefficient (ADC) map; (**D**) intravoxel incoherent motion (IVIM) pseudo-diffusion coefficient (D*) map; (**E**) IVIM perfusion fraction (f) map; and (**F**) IVIM true diffusion coefficient (D) map.

A breast consultant radiologist with over 10 years of experience reviewed lesions in the images. Lesion identification was based on hyperintense signals observed on post-contrast axial images. For each case, the region of interest (ROI) was manually delineated by tracing the largest cross-sectional area of the tumor in axial diffusion-weighted images (b-value = 800 s/mm^2^). The ROI was then manually propagated to the corresponding locations on the ADC, D, D*, and f maps. In cases with multiple malignant lesions within the same breast, only the largest lesion and its most representative axial slice were selected for analysis. Care was taken to avoid inclusion of large vessels, cystic areas, and regions of hemorrhage within the ROI, as illustrated in Figure 1.

### 2.4. Clinicopathological Assessment

Invasive breast cancers were classified according to the immunohistochemical expression of estrogen receptors (ERs), progesterone receptors (PRs), and human epidermal growth factor receptor 2 (HER2). Tumor subtypes were defined as follows:

- Luminal A: ER-positive and/or PR-positive, HER2-negative, and histologic grade 1 or 2.
- Luminal B: ER-positive and/or PR-positive with HER2 overexpression, or tumors otherwise meeting Luminal A criteria but with histologic grade 3.
- HER2-enriched: ER-negative, PR-negative, and HER2-positive.
- Triple-negative (TN): ER-negative, PR-negative, and HER2-negative. Ki-67 expression was assessed to distinguish between Luminal A and Luminal B tumors. A Ki-67 index ≥30% was considered high, indicating a more proliferative phenotype.

ER (clone SP1), PR (clone 1E2), and HER2 (clone 4B5) immunohistochemical stains were performed on formalin-fixed, paraffin-embedded (FFPE) tissue sections using the UltraVIEW DAB detection system (Ventana, FDA-approved). Tissue samples were fixed in 10% neutral-buffered formalin for a minimum of 6 h and not exceeding 72 h, in accordance with institutional protocol at King Saud University Medical City. Scoring was conducted following the ASCO/CAP 2013 guidelines:

- ER and PR:
  - Positive: ≥1% of tumor nuclei showing positive staining.
  - Negative: <1% staining of tumor nuclei.
  - Indeterminate: Applied when pre-analytic variables compromise interpretation (e.g., poor fixation or processing).
- HER2:
  - Score 0: No staining or incomplete, faint/barely perceptible membrane staining in ≤10% of tumor cells.
  - Score 1+: Incomplete, faint/barely perceptible membrane staining in >10% of tumor cells.
  - Score 2+: Incomplete and/or weak-to-moderate circumferential membrane staining in >10% of tumor cells, or complete, intense staining in ≤10% of tumor cells.
  - Score 3+: Complete, intense circumferential membrane staining in >10% of tumor cells.

### 2.5. Statistical Analysis

Statistical analyses were conducted using IBM SPSS Statistics version 25.0 (IBM Corp., Armonk, NY, USA). The Shapiro–Wilk test was applied to assess the normality of the data distributions. As the data deviated significantly from normality (*p* < 0.05), nonparametric tests were employed for group comparisons. Differences in intravoxel incoherent motion (IVIM) parameters including ADC_map, D, D*, and F between malignant and benign lesions were assessed using the Mann–Whitney U test. For comparisons across more than two subgroups (e.g., tumor grade, receptor status), the Kruskal–Wallis test was used.

To evaluate the diagnostic performance of each IVIM parameter in differentiating malignant from benign lesions, Receiver Operating Characteristic (ROC) curve analysis was performed. The area under the curve (AUC) was reported along with 95% confidence intervals (CIs), and the optimal cut-off point for each parameter was determined using Youden’s index. Corresponding sensitivity, specificity, and overall accuracy were calculated for each individual parameter. Moreover, to evaluate the diagnostic performance of IVIM parameters in combination, a binary logistic regression model was constructed using D, D*, and (f) as independent variables, with lesion diagnosis (malignant = 1, benign = 0) as the dependent variable. The predicted probabilities from this model were used to generate ROC curves. In addition, Spearman’s rank correlation coefficient (ρ) was used to assess associations between IVIM parameters and clinicopathological features, including receptor status (ER, PR, and HER2), Ki-67 index, cancer type, histologic grade, and molecular subtype. A significance threshold of *p* < 0.05 was adopted for all tests.

## 3. Results

A total of 108 lesions (73 malignant; 35 benign) were analyzed. Invasive ductal carcinoma was the most common histology (70.8%), and a majority of tumors were positive for estrogen and progesterone receptors (82.0% and 68.3%, respectively). HER2 positivity was observed in 28.8% of cases, and more than 90% of tumors were grade 2 or 3 (Table 2).

Our results showed that malignant lesions showed significantly lower diffusion metrics (ADC_map, D, D*) compared to benign lesions, indicating restricted diffusion in cancerous tissue (all *p* < 0.02). Perfusion fraction (F) did not differ significantly between groups (*p* = 0.202, Table 3).

**Figure 2:**
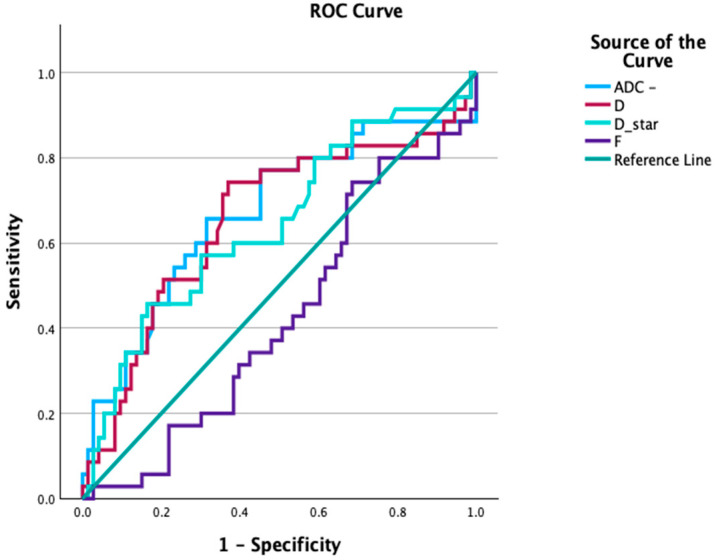
ROC curves for IVIM parameters (ADC_map, D*, F, and D) demonstrating their diagnostic performance.

The results indicated that the optimal thresholds were determined by maximizing Youden’s index for each parameter. Although ADC_map achieved perfect sensitivity, its specificity was low (11.4%). Similarly, D and D* showed high sensitivity but low specificity (<6%), while F balanced sensitivity and specificity more evenly but with slightly reduced accuracy, as seen in Table 4 and Figure 2. ADC_map, D, and D* demonstrated fair discrimination between malignant and benign lesions, with AUCs ranging from 0.64 to 0.67 (all *p* < 0.02), whereas F showed poor performance (AUC = 0.58; *p* = 0.186).

**Figure 3:**
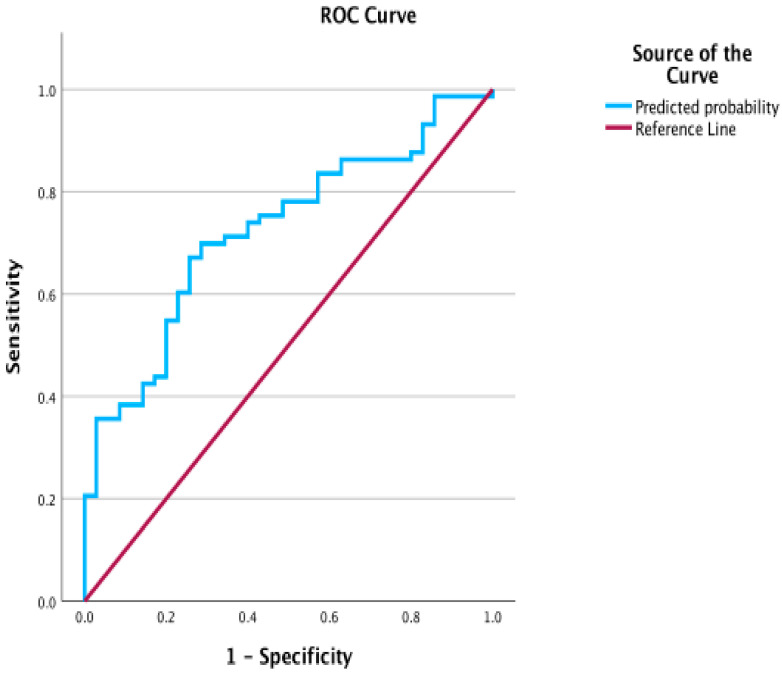
ROC curve for combined IVIM parameters (D*, F, and D).

The logistic regression model incorporating the IVIM-derived parameters D, D*, and f was statistically significant (χ^2^(3) = 13.614, *p* = 0.003), indicating that the predictors reliably differentiated between benign and malignant breast lesions (Table 5). Both D* (*p* = 0.012) and f (*p* = 0.027) contributed significantly to the model, while D was not statistically significant (*p* = 0.488). ROC analysis of the predicted probabilities yielded an AUC of 0.725 (95% CI: 0.628–0.822, *p* < 0.001). The optimal cutoff value (0.691) achieved a sensitivity of 67.1% and specificity of 74.3%, resulting in an overall classification accuracy of 70.4% (Figure 3 and Table 4). This combined model outperformed the diagnostic performance of individual IVIM parameters and ADC_map.

Across clinical and pathologic subgroups, IVIM parameters showed few meaningful differences or associations (Table 6). For hormone receptor status (ER and PR), HER2 expression, Ki-67 proliferation index, cancer type (IDC vs. ILC vs. DCIS), and luminal subtype, none of the four parameters reached statistical significance in group comparisons (all *p* ≥ 0.209) and Spearman correlations with these variables were uniformly weak (|ρ| ≤ 0.165, all *p* > 0.05). In contrast, histologic grade was associated with significant variation in both ADC_map (*p* = 0.042) and D* (*p* = 0.046), reflecting lower diffusion measures in higher-grade tumors. The corresponding correlations between grade and ADC_map (ρ = 0.219) and D* (ρ = 0.192) were modest but in the expected direction, whereas F and D neither differed by grade nor correlated meaningfully (*p* ≥ 0.173; ρ ≤ 0.036). Thus, only tumor differentiation (grade) showed a consistent relationship with diffusion-weighted parameters.

Overall, IVIM parameters showed only modest mean differences across clinical and molecular subgroups, with extensive overlap driven by large within-group variability (Table 7). For example, progesterone receptor-positive tumors had an ADC_map of 1171 ± 363 × 10^−6^ mm^2^/s versus 1075 ± 233 × 10^−6^ mm^2^/s in PR-negative cases; invasive lobular carcinomas averaged ADC_map = 1234 ± 542 × 10^−6^ mm^2^/s compared to 1110 ± 239 × 10^−6^ mm^2^/s in IDC; and grade 1 lesions showed ADC_map = 812 ± 106 × 10^−6^ mm^2^/s versus 1183 ± 385 and 1120 ± 240 in grades 2 and 3, respectively. These differences were small relative to standard deviations (often 200–600 units), highlighting that no single IVIM metric clearly distinguishes subgroups on its own.

## 4. Discussion

In this study, we presented a comprehensive analysis of the efficacy of simplified intravoxel incoherent motion (IVIM) diffusion-weighted imaging (DWI) in differentiating benign from malignant breast lesions, particularly emphasizing the roles of the diffusion coefficients (D and D*) and perfusion fraction (f). A comparison with the existing literature reveals several key similarities and contrasts that provide deeper insights into the clinical applications and limitations of IVIM. Our findings provide important context when compared with the existing literature, affirming the diagnostic value of IVIM parameters, particularly in relation to the performance of IVIM metrics and their integration with conventional imaging protocol. Beyond individual IVIM metrics, we explored a multivariable logistic regression model incorporating D, D*, and f. This combined model yielded an AUC of 0.725 (95% CI 0.628–0.822)—higher than ADC_map or any single IVIM parameter—and balanced sensitivity (67.1%) and specificity (74.3%) at the optimal cutoff. These results demonstrate that IVIM parameters, when interpreted collectively, provide added diagnostic value over univariate analysis.

In comparison with prior studies, our findings provide additional insight into the diagnostic value of diffusion-derived parameters such as IVIM and ADC measurements in the evaluation of breast cancer. Our results align with those reported by Surov et al., who emphasized the complex relationship between ADC values and tumor biology, noting that lower ADC values are characteristic of malignant tumors due to their higher cellularity and restricted water diffusion [28]. This inverse association further substantiates our observations, with malignant lesions showing significantly reduced IVIM-derived D values relative to benign counterparts. Our combined model performance is consistent with Mürtz et al. [5], who reported similar gains when IVIM metrics were analyzed jointly, reinforcing that multivariate IVIM approaches may be more clinically useful than single-parameter thresholds.

Our study highlighted a notable reduction in ADC, D, and D* values, indicative of restricted diffusion. This observation supports earlier studies that have suggested that quantifying these parameters is essential for accurate tissue characterization [29]. For instance, Yu et al., 2024, demonstrated that lower D values correlate with increased cell density and proliferation in malignant tumors, indicating that such reduced diffusion is primarily due to high cellularity and low differentiation inherent in malignant tissues, thus restricting the movement of water molecules [30]. Further supporting our findings, the metabolic and microvascular characteristics associated with breast tumors can be encapsulated through IVIM parameters. Our results observed a slight elevation of (f) values in malignant lesions, consistent with findings from Chen, who indicated that higher perfusion fractions are typically associated with neoplastic processes and increased neovascularization in tumors [31]. Similar findings by Song emphasize the clinical implication of IVIM in reflecting these physiological dynamics, noting that higher perfusion fractions are indicative of malignancy-associated neovascular patterns [31]. This observation supports the premise that IVIM can effectively capture both the microstructural and physiological aspects of breast cancer, demonstrating its utility as a non-invasive imaging biomarker in the differential diagnosis of breast lesions. Such findings reinforce the idea proposed by Liu, who noted that the use of diffusion-derived parameters may enhance the predictive capabilities of imaging approaches when integrated with traditional diagnostic methods [16].

The implications of our findings are further validated by the work of Mürtz et al. 2022 [5], who utilized simplified IVIM protocols to differentiate malignant from benign lesions effectively. Their results indicated that the addition of restricted diffusion metrics yielded diagnostic performance improvements over traditional ADC analyses alone [5]. This suggests that our study supports the continued exploration of simplified IVIM approaches, which can potentially reduce acquisition times without compromising diagnostic accuracy, reinforcing the clinical application of IVIM in standard MRI protocols for breast imaging. Moreover, our findings align with the analysis conducted by Tsvetkova et al. 2022, who concluded that IVIM parameters can assist in distinguishing malignant from benign lesions, aiding in reducing unnecessary biopsies [32]. This is particularly relevant given the wide range of malignancy probabilities associated with BI-RADS 4 lesions, where the malignancy probability can range from 2% to 95% [29] The use of IVIM may aid clinical decision-making by providing additional diagnostic information, potentially reducing the need for invasive histological biopsies in uncertain cases. However, the necessity of b-value selection in deriving accurate IVIM parameters estimations and resultant diagnostic performance is an important concern [31,33]. By adjusting our DWI sequences and employing both lower and higher b-values, we aimed to capture the full spectrum of perfusion and diffusion characteristics, echoing the findings of previous studies that underscored this methodological refinement as critical for enhancing the diagnostic capability of IVIM imaging [32]. In the current study, IVIM parameters showed only modest mean differences across clinical and molecular subgroups, with extensive overlap driven by large within-group variability. These differences were small, highlighting that no single IVIM metric clearly distinguishes subgroups on its own.

Nevertheless, the moderate Nagelkerke R^2^ (0.165) and only modest accuracy (70.4%) of our combined model indicate room for improvement—potentially via machine learning classifiers or hybrid models that fuse IVIM with DCE-MRI features. The concerns raised about the limitations of IVIM when compared with dynamic contrast-enhanced MRI (DCE-MRI) results are worthy of consideration. Rashed et al. noted that DCE-MRI typically provides higher diagnostic specificity and precision than IVIM alone [34], a notion echoed by other studies that argue that despite IVIM’s diagnostic capability, it may not yet fully replace the detailed angiographic insights provided by DCE-MRI. Therefore, our findings could synthesize these viewpoints by advocating for an integrated imaging approach utilizing both IVIM and DCE-MRI, enriching diagnostic accuracy while capitalizing on the strengths of each technique.

Although IVIM shows substantial potential, its limitations should be critically examined. As noted in the studies by Liu and Mürtz, exclusive dependence on IVIM-derived parameters can be unreliable, given the documented incidence of both false-negative and false-positive results across various tumor types [16]. It is also important to acknowledge the limitations of our study, such as its retrospective design and the potential for varying operator-dependent factors in acquiring IVIM parameters including ROI size and location. Future prospective studies with larger populations may provide more substantial evidence regarding the robustness of our findings in a clinical setting. Moreover, the performance of IVIM metrics might differ across various breast fibroglandular densities, as highlighted by Mürtz et al., 2022 [5]. Hence, further stratification based on breast composition may yield additional insights into the efficacy of IVIM across varying patient populations

## 5. Conclusions

In summary, our findings contribute to the expanding knowledge base surrounding IVIM as a novel imaging biomarker for breast lesions. The potential of IVIM to differentiate between benign and malignant lesions effectively denotes its role in non-invasive prognostication, aligning with current trends toward personalized medicine in oncology. Simplified IVIM DWI, particularly when D, D*, and f are combined in a multivariate model, improves lesion discrimination compared to individual IVIM metrics or ADC alone. While further validation through larger, multi-center trials is warranted, our results underscore the promise of simplified IVIM diffusion-weighted imaging as a critical adjunctive tool in breast cancer diagnosis, facilitating timely and accurate treatment intervention.

## Figures and Tables

**Table 1:** MRI parameters of the diffusion-weighted imaging protocol.

Imaging Sequence	Value
Axial T1-weighted (T1WI)	Standard axial anatomical imaging
STIR	Short tau inversion recovery for fat suppression
DCE-T1WI	Dynamic contrast-enhanced T1-weighted imaging
Contrast Agent	Gadolinium- DOTA (Dotarem, Guerbet, Roissy CdGCEDEX, France) Dose: 0.1 mmol/kg body weight (intravenous injection)
DWI (multi-b values)	b-values: 0, 20, 200, 500, 800 s/mm^2^
Thickness/Gap	3 mm/0.5 mm
Repetition time (TR)/Echo time (TE)	9000/60 ms
Acquisition Matrix	140 × 140
Field of View	340 × 340 mm
Total Scan Time	180 s

**Table 2:** Study population characteristics.

Characteristic	Category	N (%)
Cancer type (*n* = 65)	Invasive ductal carcinoma (IDC)	46 (70.8)
	Lobular ductal carcinoma (LDC)	13 (20.0)
	Ductal carcinoma in situ (DCIS)	6 (9.2)
ER status (*n* = 61)	Positive	50 (82)
	Negative	11 (18)
PR status (*n* = 60)	Positive	41 (68.3)
	Negative	19 (31.7)
HER2 status (*n* = 59)	Positive	17 (28.8)
	Negative	42 (71.2)
Tumor grade (*n* = 57)	Grade 1	4 (7)
	Grade 2	28 (49.1)
	Grade 3	25 (43.9)

**Table 3:** Comparison of IVIM parameters between malignant and benign lesions.

Parameter	Malignant (Mean ± SD)	Benign (Mean ± SD)	*p*-Value
ADC_map	1130.4 ± 326.5	2130.0 ± 4711.4	0.004
D*	1443.6 ± 707.8	1711.3 ± 686.8	0.016
D	972.8 ± 387.0	1227.0 ± 698.1	0.009
F	148.7 ± 104.8	117.8 ± 78.4	0.202

**Table 4:** Optimal cut-off values and diagnostic metrics for IVIM parameters (Youden’s index).

Parameter	AUC	Model Quality	Std. Error	*p*-Value	95% CI	Cutoff	Units	Sensitivity	Specificity	Accuracy
ADC_map	0.671	0.55	0.060	0.004	0.55–0.78	540.3	1 × 10^−6^ mm^2^/s	100%	11.4%	71.3%
D*	0.644	0.54	0.059	0.015	0.52–0.75	536.8	1 × 10^−6^ mm^2^/s	98.6%	5.7%	68.5%
F	0.576	0.31	0.058	0.186	0.46–0.68	38.7	1 × 10^−3^	90.4%	20.0%	68.5%
D	0.657	0.53	0.060	0.009	0.53–0.77	564.8	1 × 10^−6^ mm^2^/s	91.8%	14.3%	66.7%
Combined model (D, D*, f)	0.725	-	0.049	<0.001	0.628–0.822	0.691	-	67.1%	74.3%	70.4%

**Note:** The combined model cutoff (0.691), sensitivity, specificity, and accuracy were derived from predicted probabilities of the logistic regression model using Youden’s index. Accuracy was calculated based on the classification table using this cutoff. The combined model is based on a binary logistic regression using D, D*, and f. “Model Quality” is not directly applicable for multivariate models and was instead assessed using the Hosmer–Lemeshow goodness-of-fit test (*p* = 0.731) and Nagelkerke R^2^ (0.165). The unit column is left blank because the model outputs predicted probabilities, which are unitless.

**Table 5:** Logistic regression results.

Variable	B (SE)	Wald	*p*-Value	Exp(B)
D*	−0.001 (0.000)	6.338	0.012	0.999
f	0.009 (0.004)	4.912	0.027	1.009
D	0.000 (0.000)	0.482	0.488	1.000
Constant	1.721 (0.700)	6.049	0.014	5.588

Nagelkerke R^2^ = 0.165; Hosmer–Lemeshow test (χ^2^(8) = 5.246, *p* = 0.731).

**Table 6:** Nonparametric test results and Spearman correlations of IVIM parameters across clinical and pathologic subgroups.

Variable	ADC_Map *p*-Value	ADC_Map r	D* p-Value	D* r	F *p*-Value	F r	D *p*-Value	D r
ER	0.61	0.06	0.77	0.03	0.56	−0.07	0.31	0.13
PR	0.89	0.01	0.79	−0.03	0.65	−0.05	0.58	0.07
HER2/Neu	0.22	−0.16	0.20	−0.16	0.68	0.05	0.13	−0.19
Ki-67	0.40	−0.12	0.21	−0.17	0.30	−0.14	0.41	0.11
Cancer type	0.94	0.10	0.37	0.03	0.82	−0.09	0.71	0.12
Luminal subtype	0.76	0.10	0.74	0.03	0.595	−0.09	0.77	0.12
Histologic grade	0.04 *	0.21	0.04 *	0.19	0.19	0.03	0.17	0.22

* Statistically significate.

**Table 7:** IVIM parameter means ± SDs across clinical and pathologic subgroups.

Parameter	Category	ADC_Map	D*	D (Mean ± SD)	F (Mean ± SD)
ER status	Positive	1141.43 ± 345.33	1407.57 ± 488.75	981.14 ± 400.38	144.22 ± 82.76
ER status	Negative	1119.44 ± 240.02	1372.22 ± 319.56	1016.19 ± 218.44	115.53 ± 51.62
PR status	Positive	1171.19 ± 363.35	1406.81 ± 399.41	1026.89 ± 420.07	139.24 ± 78.82
PR status	Negative	1074.68 ± 232.69	1387.72 ± 555.40	919.82 ± 242.25	135.98 ± 77.77
Cancer type	IDC	1110.19 ± 238.67	1329.42 ± 336.52	973.82 ± 279.96	133.10 ± 70.38
Cancer type	ILC	1233.99 ± 541.90	1658.78 ± 714.04	1043.02 ± 610.14	156.28 ± 102.46
HER2 status	Positive	1160.53 ± 233.21	1431.79 ± 298.16	1016.43 ± 204.58	137.03 ± 70.17
HER2 status	Negative	1123.49 ± 367.20	1382.33 ± 526.29	973.21 ± 434.83	138.65 ± 82.59
Ki-67 level	Low	1180.63 ± 422.65	1499.17 ± 613.73	1014.34 ± 506.62	150.99 ± 96.30
Ki-67 level	High	1103.79 ± 226.79	1325.62 ± 276.37	969.38 ± 222.23	128.38 ± 60.22
Luminal	Luminal A	1162.90 ± 389.15	1384.95 ± 414.09	1022.43 ± 458.24	139.37 ± 83.42
subtype	Luminal B	1098.51 ± 246.07	1452.80 ± 633.19	898.55 ± 246.48	153.91 ± 84.56
	HER2-enriched	1144.39 ± 243.81	1438.25 ± 268.08	1032.58 ± 227.53	124.35 ± 47.38
Tumor grade	Grade 1	811.50 ± 106.21	921.15 ± 58.19	651.30 ± 274.50	105.26 ± 81.24
Tumor grade	Grade 2	1183.00 ± 385.39	1457.80 ± 558.98	1038.94 ± 462.44	136.89 ± 82.30
Tumor grade	Grade 3	1119.80 ± 239.99	1386.52 ± 305.96	968.57 ± 221.02	142.83 ± 75.14

## Data Availability

The data presented in this study are available on request from the corresponding author.
